# Fatigue Failure from Inner Surfaces of Additive Manufactured Ti-6Al-4V Components

**DOI:** 10.3390/ma14040737

**Published:** 2021-02-05

**Authors:** Joel de Jesus, José António Martins Ferreira, Luís Borrego, José D. Costa, Carlos Capela

**Affiliations:** 1 Centre for Mechanical Engineering, Materials and Processes (CEMMPRE), Department of Mechanical Engineering, University of Coimbra, 3004-531 Coimbra, Portugal; martins.ferreira@dem.uc.pt (J.A.M.F.); borrego@isec.pt (L.B.); jose.domingos@dem.uc.pt (J.D.C.); carlos.capela@ipleiria.pt (C.C.); 2 Department of Mechanical Engineering, Coimbra Polytechnic—ISEC, Rua Pedro Nunes, 3030-199 Coimbra, Portugal; 3 School of Technology and Management, Polytechnic Institute of Leiria, 2411-901 Leiria, Portugal

**Keywords:** additive manufacturing, fatigue, TiAl6V4 alloy, fatigue life prediction

## Abstract

Selective laser melting (SLM) is an additive manufacturing process for producing metallic components with complex geometries. A drawback of this process is the process-inherent poor surface finish, which is highly detrimental in materials submitted to fatigue loading situations. The goal of this work is to analyze the fatigue behavior of Ti-6Al-4V specimens with internal axial channels under the following different conditions: hole drilled, hole as manufactured, and hole threaded M4 × 0.7. All the cases studied showed a lower fatigue performance as compared with solid samples due to the surface roughness and geometry effect that produced a surface stress concentration leading to a reduction in fatigue strength. The fractography revealed that crack initiation occurred from the internal surface in all specimens with internal channel mostly from defects as unfused particles and lack of fusion zones, while for the solid specimens crack initiation was observed from the external surface due to insufficient fusion defect. The application of the Smith-Watson-Topper energy-based parameter was revealed to be a good tool for fatigue life prediction of the different series studied.

## 1. Introduction

Selective laser melting (SLM) is one additive manufacturing (AM) technology that is increasingly used in production in specific applications, namely, it is capable of producing complex components through layer deposition. In addition, it provides a high degree of design freedom, can optimize and integrate functional features, and can manufacture small batch sizes at reasonable unit costs. Usually, SLM parts are submitted to a post heat treatment in order to eliminate detrimental residual stresses induced during the manufacturing process due to high temperature gradients [1]. One of the materials frequently used and studied in the SLM process is the titanium Ti-6Al-4V alloy, due to its qualities as a light alloy characterized by having excellent mechanical properties and corrosion resistance combined with low specific weight; Ti-6Al-4V is commonly used in aerospace, biomedical, and other high-performance engineering applications as reported by Petrovic et al. [2] and Mur et al. [3]. Regarding the automotive and aerospace industries, the AM technique leads to weight reduction, as well as decreased energy use and material waste, as indicated by Guo and Leu [4] and Frazier [5]. Xuan and Nastac [6] obtained an increase in fatigue behavior of Ti-6Al-4V parts produced by SLM when submitted to stress relief treatment as compared with as-built parts.

Although titanium Ti-6Al-4V alloy has benefits, the machinability of titanium alloys manufactured by the conventional processing technologies shows some drawbacks such as sticking, blade wear-out, wear of the tools, waste of raw material, and slow production times. These aspects have led to a higher utilization of powder-bed based metal additive manufacturing technologies, i.e., selective laser melting (SLM), as well as electron beam melting (EBM) especially in complex components, namely, with internal channels. Components with internal channels have enumerable applications such as dental implants, cooling channels in turbine blades, and cooling channels in valves.

The monotonic properties of additively manufactured material can be equated with conventionally processed alloys [7]; however, in applications where the components are submitted to cycle loadings the main process inherent defects such as porosity and rough surface are detrimental to the fatigue behavior. Surface roughness plays an important role in fatigue crack initiation. Its influence on fatigue performance has been investigated for components produced by AM in TiAl6V4 alloy.

Edwards and Ramulu [8] studied the fatigue performance of flat bars produced by SLM with Ti-6Al-4V alloy and concluded that the fatigue strength was 75% lower as compared with that in wrought material. The detrimental effects of residual porosity, surface roughness, and residual stress were responsible for this behavior in as-built components. Wycisk et al. [9] observed that, for high cycle fatigue tests (R = 0.1), the fatigue properties of laser additive manufactured Ti-6Al-4V in polished specimens were more superior than the material in an as-built condition, due to the process inherent high surface roughness. Greitemeier et al. [10] showed that an increase in fatigue performance (R = 0.1) in samples produced by SLM with Ti-6Al-4V submitted to hot isostatic pressing was not achieved due to fatigue cracks that were initiated at the rough as-built surface. A surface modification by milling resulted in a significant improvement of the fatigue strength. Chan et al. [11] reported that the fatigue life of Ti-6Al-4V decreased with increasing maximum surface roughness acting as multiple stress concentration points at the surface features. Kasperovich and Hausmann [12] and Chern [13] found similar results, recommending the application of hot isostatic pressing and surface treatments (i.e., machining, polishing, among others) for increasing the fatigue performance. Globally, there is agreement that the application of hot isostatic pressing to reduce residual internal defects and to introduce compressive residual stress on the surface alloy with a good surface finish is the best option for increasing the fatigue performance of laser additive manufactured Ti-6Al-4V.

Konolvalov et al. [14] performed fatigue tests on commercially pure titanium VT1-0 and revealed that electropulse treatment of samples in the mid-stage of tests enhanced the fatigue life of the material by ≈1.3 times as compared with samples without this treatment. Nevertheless, a good surface finish through machining is the most inexpensive and efficient solution for increasing the fatigue performance of laser additive manufactured Ti-6Al-4V.

The build thickness may have an influence on the final surface roughness of components produced by powder-bed based metal additive manufacturing technologies. Razavi et al. reported that a lower build thickness of EBM parts resulted in higher surface roughness [15]. This aspect is important when components with internal channels and lower thickness are built. Günther et al. [16] studied the effect of internal channels and surface roughness on the high cycle fatigue behavior of Ti-6Al-4V processed by SLM and concluded that components with internal channels were characterized by a slightly lower fatigue limit as compared with as-built solid samples. Furthermore, the fracture surface revealed crack initiation frequently at the rough internal channel surface. Fatigue behavior is strongly affected by surface roughness and porosity. Finally, the same authors also described that increasing the inner diameter reduced the fatigue strength.

In this work, fatigue tests were performed for a stress ratio of R = 0 (ratio between the minimum stress and maximum stress), with the main objective of analyzing the effects of surface roughness and stress concentrations on the fatigue response of titanium alloy Ti-6Al-4V components with inner surfaces manufactured by SLM.

## 2. Materials and Methods

**Figure 1:**
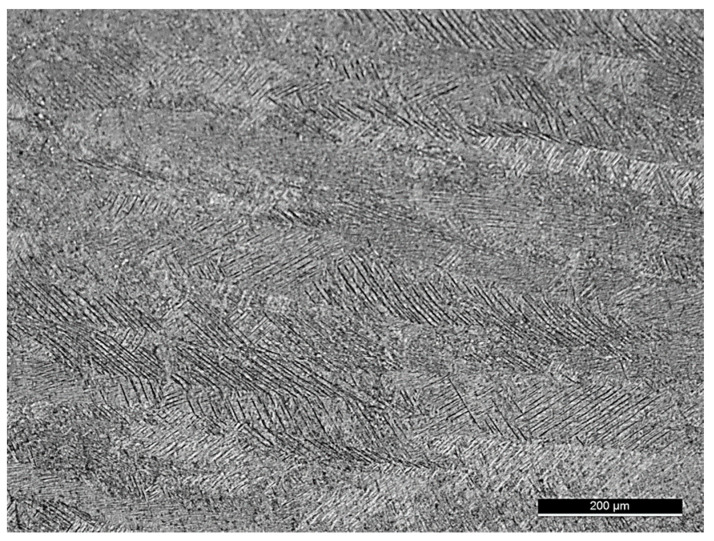
Microstructure of the specimens.

Experimental tests were performed using dog bone round specimens, synthesized by Lasercusing^®^ (3D Systems, Rock Hill, SC, USA), with layers growing towards the direction of loading application, incorporating longitudinal inner holes to simulate internal channels. The specimens were produced using a ProX DMP 320 (3D Systems, Rock Hill, SC, USA) high-performance metal additive manufacturing system, incorporating a 500 w fiber laser. The metal powder used was the titanium Ti-6Al-4V Grade 23 alloy (Osprey^®^ Metal Powder, Sandviken, Sweden), with a chemical composition, according to the manufacturer indicated in Table 1. After the specimens were manufactured, they were subjected to a heat treatment, for the purpose of reducing residual stresses, which consisted of slow and controlled heating to 670 °C, followed by maintenance at 670 °C ± 15 °C for 5 h, and finally by cooling to room temperature in air [17]. The microstructure of the material samples was observed using a Leica DM4000 M LED (Leica Microsystems, Wetzlar, Germany) optical microscope after etching with Kroll’s reagent (6% H_2_NO_3_, 1% HF, and 93% H_2_O), as suggested in ASTM E40725 standard, presenting Figure 1 a representative image. Figure 1 shows a microstructure with an acicular morphology where two phases are identified, i.e., material with a martensitic phase α (or α′), due to the fast solidification, quite similar to that observed by Greitmeier et al. [18]. The observation of the figure also shows the formation of long grains in the deposition plane and the transitions between layers. The mechanical properties, obtained by tensile testing in previous work, are an ultimate tensile strength of 1147 MPa and a Young’s modulus of 126 GPa [19].

**Figure 2:**
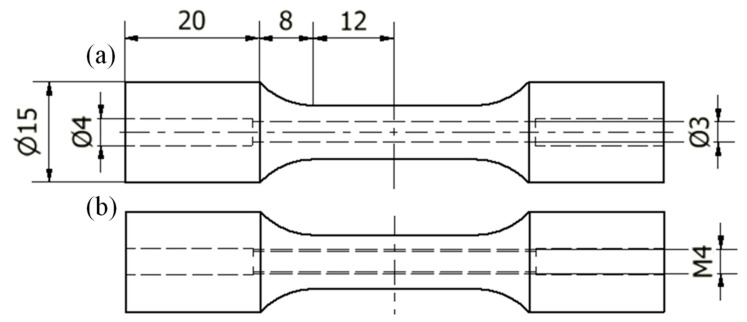
Geometry and dimensions of the holed specimens. (**a**) drilled specimens and (**b**) threaded specimens (M4 × 0.7).

Figure 2 shows the geometry and dimensions of the specimens used. Figure 2a shows the holed specimens and Figure 2b presents the internally threaded specimens (M4 × 0.7). Moreover, specimens without hole were produced which had the same external geometry and dimensions. All specimens were produced with 8 mm of external diameter in the prove zone. The holed specimens were produced through two different processes, one directly by SLM and the other by drilling at 800 rpm of rotation speed and 2 mm/min with lubrication. The specimens with internal thread were produced by electrical discharge machining (EDM).

The experimental fatigue tests were performed using an INSTRON servohydraulic (Instron, Norwood, MA, USA), closed-loop mechanical test machine with 100 kN load capacity, interfaced to a computer for machine control and data acquisition. All tests were conducted in air, at room temperature, at a frequency of 20 Hz and the stress ratio R = 0 [21]. The fracture surfaces were observed using a Leica DM4000 M LED (Leica Microsystems) optical microscope and analyzed by SEM using a Philips XL 30 scanning electron microscope (Philips, Eindhoven, The Netherlands). The roughness evaluation was carried out according to DIN EN ISO 4288 [22] standard using a rugosimeter Mitutoyo, Surftest SJ-500 (Mitutoyo, Kawasaki, Japan) along external and internal surfaces.

The outcomes considered were the different S-N curves obtained through the fatigue tests and the roughness evaluation through the parameters: roughness average Ra, maximum peak-to-valley height Ry, average maximum peak-to-valley of ten consecutive sampling lengths within the measuring length Rz, and the average spacing of adjacent peaks in the surface profile Dp.

## 3. Results

Table 2 presents the roughness parameters measured for each case analyzed. The polishing process, applied to the specimens without hole, produced lower values of R_a_, R_y_ and R_z_ as comparing with the internal surface finish for the other cases, i.e., hole drilled specimens, hole as manufactured, and internal surface of thread, which meant a good surface finish of external surfaces. This polish process was applied to all specimens for all external surfaces in order to understand the influence of inner surface roughness on fatigue behavior. On the one hand, the worst surface finish was found in the thread internal surface for the specimens with internal thread followed by the series with the hole as manufactured. On the other hand, the best surface finish among these three series was the hole drilled series. It is important to note that the roughness of thread internal surface for the specimens with internal thread were measured on the surfaces with the larger diameter.

Average elastic stress concentration factor k_ts_, introduced by multiple micro-notches due to surface roughness, can be estimated by Equation (1) according to Arola and Williams [23] as follows:

(1) $$k_{\mathrm{ts}}=1+n\left(\frac{R_{a}}{D_{p}}\right)\left(\frac{R_{y}}{R_{z}}\right)$$

where R_a_ is the roughness average, R_y_ the maximum peak-to-valley height, R_z_ the average maximum peak-to-valley of ten consecutive sampling lengths within the measuring length, D_p_ the average spacing of adjacent peaks in the surface profile, and the n parameter equal to 2 for uniform stresses.

Table 3 presented the average elastic stress concentration factor due to the surface finish k_ts_ and the dynamic stress concentration factor (the latter to be commented on later). It can be observed that the higher average elastic stress concentration factor was observed in the specimens with the hole as manufactured. The average elastic stress concentration factor due to the surface finish shows the following values: as AM (without hole) < hole drilled < internal surface of thread < hole as manufactured. The average elastic stress concentration factor showed in Table 1 considers only the surface finish and not the geometric effect. In the hole drilled and hole as manufactured series, the geometric effect is reduced and does not affect the elastic stress concentration factor because the load was applied longitudinally to the hole, whereby the surfaces roughness was the only cause for the stress concentration. For the internal thread specimens, it is necessary to include the geometric effect due to the thread itself.

In order to estimate the elastic concentration factor due to geometry k_tg_, Equation (2) can be used as follows:

(2) $$k_{\mathrm{tg}}=\frac{\sigma_{\max}}{\sigma_{n}}$$

where σ_max_ is the maximum stress at the notch root and σ_n_ is the nominal stress.

**Figure 3:**
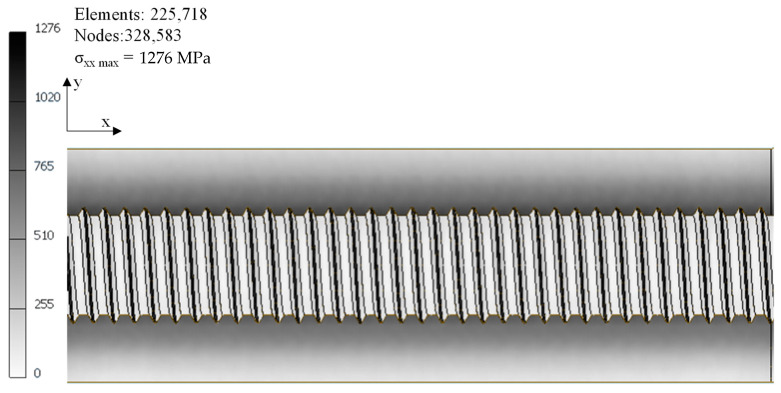
FEM results, σ_max_ = 1276 MPa.

The maximum stress σ_max_ was estimated by the finite element method (FEM) using the CAD software Autosesk Inventor^®^ (Autodesk Inc., Marin County, CA, USA), assuming an isotropic linear elastic material (E = 126 GPa). This software can create an accurate model of the geometry of specimens with internal thread as well as similar loading direction. The load (10 kN) was applied on the *x*-axis in the direction represented in the Figure 3. The nominal stress, σ_n_, was obtained by dividing the maximum applied load (10 kN) by the remaining cross-section area. Figure 3 shows the FEM results, which estimated that σ_max_ = 1276 MPa. Applying Equation (2), k_tg_ = 4.711 was obtained. As clearly observed, the stress concentration was verified in the internal surface of thread (darker zones). Finally, the total elastic concentration factor for the specimens with internal thread can be calculated by multiplying k_tg_ by k_ts_ (Table 3) obtaining k_t_ = 5.936. 

Finally, the total elastic stress concentration factor shows the following values: as AM (without hole) < hole drilled < hole as manufactured < hole threaded. As expected, these results show that the specimen series most affected by the stress concentration is the series with the hole threaded.

**Figure 4:**
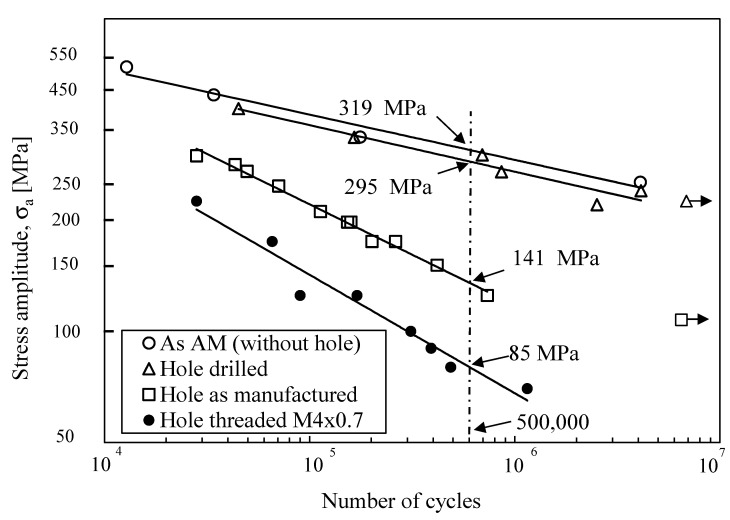
Comparison of S-N curves.

The fatigue strength for all series is plotted in Figure 4, in terms of S-N curves. As expected, the fatigue strength is strongly influenced by the internal surface roughness and the geometry that generates stress concentration, which induces much lower fatigue strength for specimens with internal thread, caused by the roughness and geometry effect. The dynamic stress concentrations, k_f_, were calculated using Equation (3) as follows:

(3) $$k_{f}=\frac{\sigma_{\mathrm{fu}}}{\sigma_{\mathrm{fe}}}$$

where σ_fu_ is the fatigue strength for smooth specimens and σ_fe_ is the fatigue strength for specimens with stress concentration.

The fatigue strength for smooth specimens and the fatigue strength of each series estimated was at 5 × 10^6^ cycles. The values of k_f_, from Table 3, are plotted and show a strong reduction in fatigue endurance of the specimens with internal thread followed by the specimens with the hole as manufactured. Samples with internal drilled channels are characterized by only a slightly lower fatigue strength as compared with specimens without channels, i.e., solid specimens.

**Figure 5:**
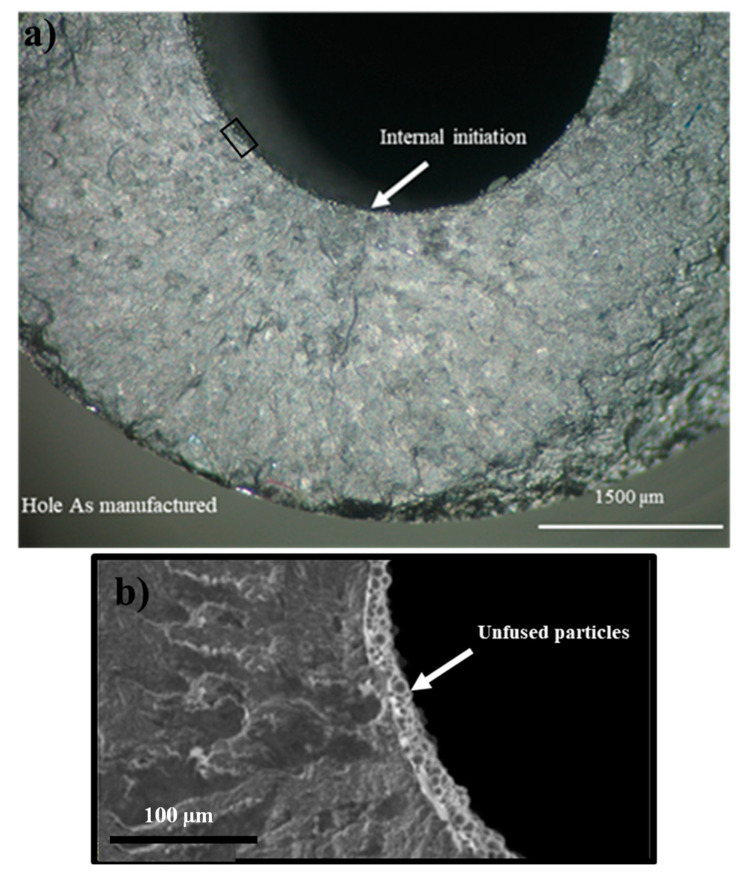
Fracture surface of hole as manufactured, σ_a_ = 175 MPa. (**a**) Crack initiation; (**b**) Unfused particles.

**Figure 6:**
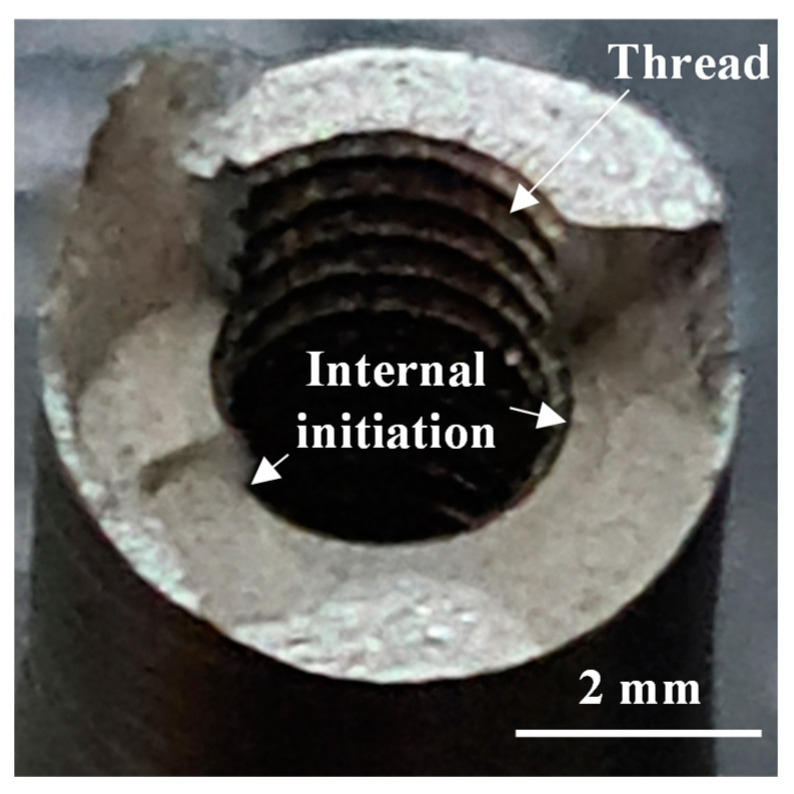
Fracture surface of hole threaded, σ_a_ = 80 MPa.

**Figure 7:**
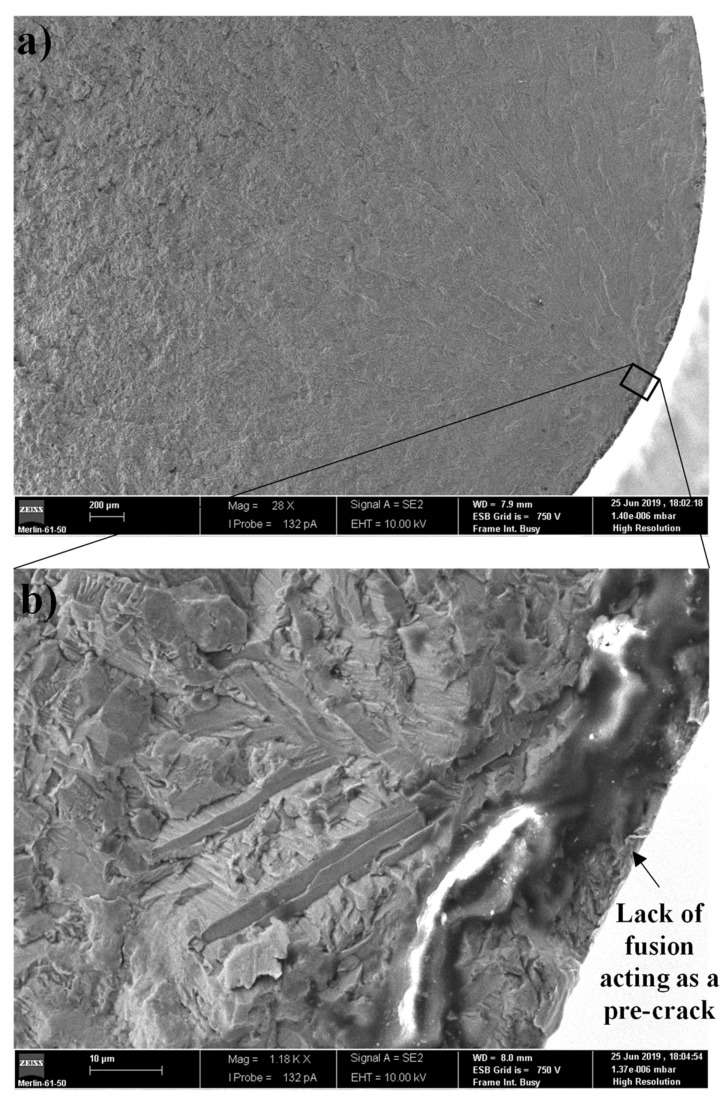
Fracture surface of solid specimen, σ_a_ = 350 MPa. (**a**) Fracture surface; (**b**) Crack initiation site.

Representative fracture surfaces of the hole as manufactured, hole threaded, and without hole series are presented in Figure 5, Figure 6 and Figure 7, respectively. The crack initiation occurred from the internal surface in all specimens with internal channel mainly from defects such as lack of fusion/unfused particles (see representative example in Figure 5 and Figure 7). The hole as manufactured series showed a high number of unfused particles, as observed in Figure 5b, increasing the surface roughness as well as the stress concentration, while the hole drilled series presented lower quantity of defects, and therefore presented a lower roughness in the internal surface. The hole threaded series showed a better surface finish in the internal surface of the thread, the EDM process remove the unfused particles but, nevertheless, the crack initiation always occurred from the internal surface of thread due to the stress concentration.

Figure 7a shows a smoother fracture surface, typical of a fatigue test performed with a stress amplitude in the elastic regime. The initiation of the crack occurred on the surface of the specimens, from a single critical point where, normally, there was a lack of fusion defect, as can be seen in Figure 7b (20× magnification of Figure 7a).

Predictions of the fatigue life were achieved using the energy-based SWT parameter, this parameter has been successfully applied in different situations [24,25,26,27] using Equation (4) as follows:

(4) $$\mathrm{SWT}=\sigma_{\max}\cdot \varepsilon_{a}$$

**Figure 8:**
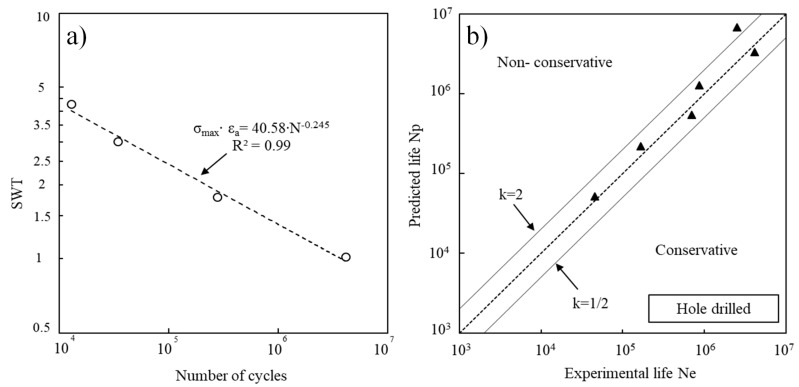
(**a**) SWT parameter vs. number of cycles to failure for the solid specimens; (**b**) Fatigue life predictions vs. experimental fatigue lives for the hole drilled series.

Figure 8 shows the SWT parameter versus the number of cycles to failure (Figure 8a) and fatigue life predictions vs. experimental fatigue lives for the hole drilled series (Figure 8b). This prediction life method involved estimating the curve of the SWT parameter depending on the number of cycles for solid specimens, and then calculating the SWT parameter, considering an elastic regime and the elastic stress concentration (k_t_) for each case, and then replacing it for each series in the equation presented in Figure 8a to calculate the corresponding number of cycles. There is a good correlation between predicted and experimental results. Indeed, 83.3% of the results for the hole drilled specimens are within scatter bands with a factor of 2.

**Figure 9:**
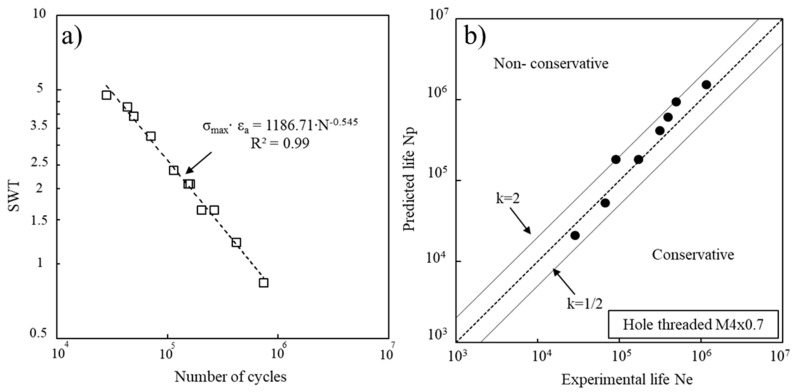
(**a**) SWT parameter vs. number of cycles to failure for the hole as manufactured series; (**b**) Fatigue life predictions vs. experimental fatigue lives for the hole thread series.

The method described previously was used to predict the fatigue life of the specimens with internal thread but unsuccessfully, because, as observed in Figure 4, the slope of the S-N curve for specimens without hole is lower than the hole drilled and hole thread series, moreover, the equation indicated in Figure 8a cannot be successfully applied. Therefore, in order to apply the prediction method, a new curve SWT vs. N_f_ (Figure 9a) was plotted, which belongs to the hole as manufactured series due to the similar slope of the S-N curve between both series, hole as manufactured and hole threaded. Figure 9b shows a good correlation between predicted and experimental results because 100% of the points are within scatter bands with a factor of 2.

## 4. Discussion

As described previously, fatigue behavior is strongly affected by internal surface roughness, mainly in components manufactured by SLM. A reduction in the fatigue strength was observed in each of the studied series as follows: a reduction of 8.5% for the hole drilled series, 226% for the hole as manufactured series, and 375% for the hole threaded M4 × 0.7 series (all values obtained from Figure 3, at 5 × 10^5^ cycles). As compared with solid specimens, the surface roughness is the main cause of this fatigue strength reduction. In the hole drilled series, the drilling process did not leave unfused particles as they were removed from the channel produced directly by SLM. The sticking phenomenon of machine drilling softened the surface roughness induced by characteristic defects of the SLM process due to a lack of fusion. Although there are beneficial effects from the channels produced by machine drilling that lower the production time, the recognized drawbacks seem to be tool wear, the cost of another process, and tool fracture inside of the channel. The hole as manufactured series showed a strong reduction in fatigue strength (226%) due to internal surface roughness which was full of unfused particles and lack of fusion zones; similar results were found by Günther et al. [16]. The greatest reduction in fatigue strength was found in the hole threaded series, due to the effect of geometry and the poor surface finish of the internal surface of the thread.

The geometry effect was registered only for the hole threaded series. All cited defects caused stress concentration in the loading surface, due to the surface finish/geometry effect, in the following order: without hole < hole drilled < hole as manufactured < hole threaded, which explained the fatigue strength reduction. The high values of stress concentration verified in the hole as manufactured series and the hole threaded series made the slope of the S-N curves higher, which meant that, in those tests, the crack initiation periods were lower than the as manufactured (without hole) and hole drilled series.

Crack initiation occurred from the internal surface in all specimens with internal channel mostly from defects such as lack of fusion (hole drilled series) and unfused particles/lack of fusion zones (hole as manufactured and hole threaded series), while for the for solid specimens the crack initiation was observed from the external surface (as expected) due to insufficient fusion defects.

Application of an SWT energy-based parameter revealed that it was a good tool for predicting the fatigue life of the different series studied, given that predicted and experimental points were practically all within scatter bands with a factor of 2.

## 5. Conclusions

A methodical study was carried out on the fatigue behavior of Ti-6Al-4V components regarding the effect of inner surfaces, all initially manufactured by selective laser melting. The comparison was performed by as-built and also drilled or electrical discharge machined inner surfaces. The results of the analysis of surface roughness and stress concentration effects on the fatigue response allowed us to make the following conclusions:

- The stress concentration due to surface roughness associated with the stress concentration (internal thread) derived from the geometry of inner surfaces caused a strong fatigue strength reduction.
- A good fatigue performance was obtained for internal surfaces with better finishing.
- The Ti-6Al-4V alloy showed high notch sensibility.
- Internal surfaces produced by SLM showed significant unfused particles and a lack of fusion defects that led to high surface roughness and lower fatigue strength, while drilling surfaces showed better surface finish and, consequently, higher fatigue strength.
- The crack initiation occurred from defects such as lack of fusion and unfused particles/lack of fusion zones, and crack initiation was accelerated by the presence of these defects.
- Application of an SWT energy-based parameter was shown to be an adequate tool for predicting the fatigue life of the material and geometrical conditions analyzed.

## Figures and Tables

**Table 1:** Chemical composition of the titanium Ti-6Al-4V alloy (wt.%) [20].

Al	V	O	N	C	H	Fe	Ti
5.50–6.50	3.50–4.50	<0.15	<0.04	<0.08	<0.012	<0.25	Balance

**Table 2:** Roughness parameters for each case.

Series	Roughness	Average	Standard Deviation
As AM (without hole)	R_a_ (µm)	0.1665	±0.0228
	R_y_ (µm)	1.4451	±0.1236
	R_z_ (µm)	1.1897	±0.0895
	D_p_ (µm)	46.325	±3.2594
Hole drilled	R_a_ (µm)	0.9781	±0.1117
	R_y_ (µm)	8.9417	±1.6472
	R_z_ (µm)	8.7894	±1.6472
	D_p_ (µm)	18.361	±8.6816
Hole as manufactured	R_a_ (µm)	6.9825	±1.2576
	R_y_ (µm)	51.121	±2.597
	R_z_ [µm]	42.442	±2.548
	D_p_ [µm]	7.862	±1.6566
Internal surface of thread	R_a_ [µm]	8.448	±1.2577
	R_y_ [µm]	71.335	±2.499
	R_z_ [µm]	52.251	±2.450
	D_p_ [µm]	88.356	±9.2594

**Table 3:** Stress concentration factors.

Series	K_ts_	K_f_
As AM (without hole)	1.008	-
Hole drilled	1.120	1.090
Hole as manufactured	3.382	3.190
Internal surface of thread	1.261	5.884

## Data Availability

The data presented in this study are available on request from the corresponding author. The data are not publicly available due to privacy.
